# Handlungsempfehlungen bei oraler Candidose unter Biologikatherapie bei Psoriasis – Ergebnisse eines Expertenmeetings

**DOI:** 10.1007/s00105-024-05410-y

**Published:** 2024-09-03

**Authors:** Katharina Meier, Stefan Beissert, Kamran Ghoreschi, Sandra Philipp, Beate Schwarz, Hans-Jürgen Tietz, Khusru Asadullah

**Affiliations:** 1 Klinik für Dermatologie, Venerologie und Allergologie, Charité – Universitätsmedizin Berlin, Campus Charité Mitte, Luisenstr. 2, 10117 Berlin, Deutschland; 2 Klinik und Poliklinik für Dermatologie, Universitätsklinikum Carl Gustav Carus Dresden, Dresden, Deutschland; 3 Hautarztpraxis Dr. med. Friedrich/Dr. med. Philipp, Oranienburg, Deutschland; 4 Hautarztpraxis/Studienzentrum Dr. Schwarz, Langenau, Deutschland; 5 mycoclinic, Institut für Pilzkrankheiten und Innere Medizin, Berlin, Deutschland; 6 Dermatologie Potsdam MVZ, Potsdam, Deutschland

Moderne Systemtherapien haben in den letzten 10 Jahren die Behandlung der Psoriasis revolutioniert, sodass als Therapieziel die Erscheinungsfreiheit gilt bei einem insgesamt sehr guten Sicherheitsprofil. Dennoch kann es, insbesondere unter einer Anti-Interleukin-17-Therapie, zu oralen Candidosen kommen. Ein Therapieabbruch ist in der Regel nicht notwendig, da orale Candidosen gut behandelbar sind. Um diagnostische und therapeutische Unsicherheiten bezüglich der Candidosen unter Biologikatherapie zu vermeiden, wurden diese Handlungsempfehlungen erarbeitet.

Etwa 2–4 % der Weltbevölkerung leiden unter Psoriasis, einer chronisch-entzündlichen Erkrankung, die sich primär an der Haut manifestiert [9]. Zu den Auswirkungen auf die Lebensqualität der Patienten tragen Komorbiditäten wie die Psoriasisarthritis sowie psychischer Stress bei. Insbesondere mit monoklonalen Antikörpern gegen Interleukin (IL)-23 und IL-17, die als zentrale Treiber der Entzündungsaktivität identifiziert wurden, ist die Psoriasis jedoch inzwischen bis zur Komplettremission therapierbar [5]. Bei der Therapiewahl müssen individuelle Komorbiditäten und medikamentenspezifische Nebenwirkungsprofile bedacht werden. IL-17 übernimmt neben der zentralen Rolle in entzündlichen Erkrankungen wie der Psoriasis oder Psoriasisarthritis auch eine Schlüsselrolle bei der Abwehr mukokutaner Candidosen indem es die Chemotaxis von Neutrophilen und die antimikrobielle Peptidaktivität induziert [8]. Im Rahmen einer antiinflammatorischen IL-17-Inhibition kann es daher zu mukokutanen Pilzinfektionen, zumeist Candidosen kommen (Abb. 1). Diese sind selten chronisch-rezidivierend oder schwer verlaufend.

**Abb. 1:**
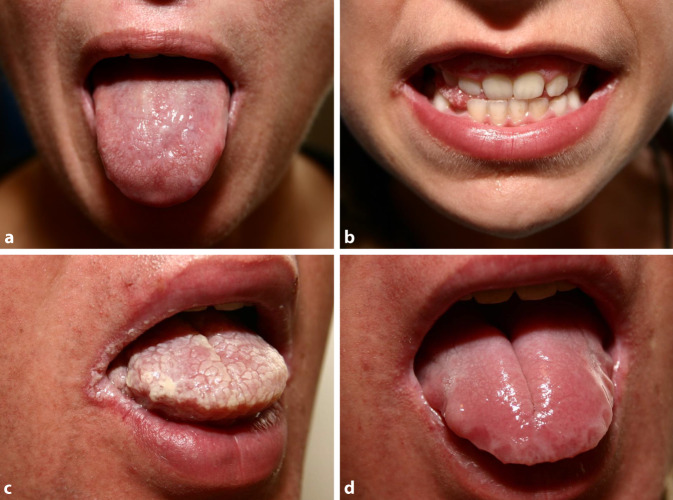
Klinische Bilder der oralen Candidose mit weißlichen, zum Teil abstreifbaren Belägen der Zunge (**a**), weißlich belegt Mundwinkelrhagaden (**b**), ausgeprägte weiße papillomatöse Beläge der Zunge und der Mundwinkel (**c**) und dezente Papillenhypertrophie der Zunge (**d**) mit deutlichem Abdruck der Zahnleiste. (Aus [11], © H.-J. Tietz, mit freundl. Genehmigung)

Die Symptome können folgende sein:

- Rötung der Schleimhaut,
- weißliche Beläge, abstreifbar,
- Mundtrockenheit, Brennen auf der Zunge,
- pelziges Mundgefühl, Geschmacksstörungen,
- bei sehr ausgeprägten Formen: Schmerzen und Probleme beim Essen, Trinken, Schlucken.

Sowohl der Wirkmechanismus als auch die Bindungsaffinität der IL-17-Inhibitoren scheinen einen Einfluss auf die therapeutische Wirksamkeit bei der Psoriasis wie auch auf die Inzidenz von *Candida*-Infektionen zu haben [10]. So weist Bimekizumab als dualer Inhibitor von IL-17A und IL-17F eine höhere Candidoseinzidenz auf als z. B. Secukinumab, das mit einer geringeren Affinität nur an IL-17A bindet. In klinischen Studien zur Therapie der Psoriasis mit Bimekizumab lag die Inzidenz oraler Candidosen bei 7,3 % [2]. Eine orale Candidose unter der IL-17-Inhibition zeigt zumeist eine leichte bis mittelschwere Symptomlast (Klinik s. auch Abb. 1), ist lokal auf Schleimhäute und Haut begrenzt und führt nicht zu einer Exazerbation der Psoriasis [10].

## Candidosen als Folge effektiver Immunmodulation

Eine Kolonisation von Haut und Schleimhaut mit *Candida species* bei Patienten mit Psoriasis ist nicht selten [4]. Einige Patienten sind aufgrund von Risikofaktoren empfänglicher für Pilzinfektionen als andere (Tab. 1; [4]). Als häufigster Pilz im Zusammenhang mit einer Anti-IL-17-Therapie wurde *Candida albicans* identifiziert, aber auch Vertreter anderer *Candida* Spezies und Dermatophyten wurden gefunden. Als kommensaler opportunistischer Hefepilz kommt *Candida* in der normalen Mikroflora der Haut sowie auf Schleimhäuten vor. Bei Candidosen unter einer IL-17-Therapie handelt es sich primär um oropharyngeale Infektionen. Kutane Infektionen kommen selten vor und vulvovaginale scheinen einem anderen Pathomechanismus zu unterliegen, bei dem u. a. lokale Östrogenspiegel sowie eine IL-1β-induzierte Hyperinflammation maßgeblich zur Pathogenese beitragen [3]. Voraussetzung für eine oropharyngeale *Candida*-Infektion ist die Anwesenheit von *Candida albicans* in der Mundschleimhaut. Daher gilt es, präventive Maßnahmen zu ergreifen. Sollte es dennoch zu einer Infektion kommen, lässt sich diese meist gut behandeln und führt nicht zum Abbruch der sonst wirksamen Psoriasistherapie. In den gängigen Leitlinien werden unterschiedliche Therapieschemata empfohlen; die sich aber nicht explizit auf Patienten mit oraler Candidose infolge einer antientzündlichen Therapie beziehen [5, 7].

**Tab. 1:** Risikofaktoren für die Entwicklung einer Candidose

Alter ≥ 51 Jahre
Diabetes mellitus
Tragen von Zahnspangen und Prothesen
Rauchen
Therapie mit Immunsuppressiva oder Breitbandantibiotika
Angeborene Immundefekte, z. B. in der Interleukin-17-Achse
Infektion mit dem Humanen Immundefizienz-Virus (HIV)
Anhaltende Neutropenie
Intraabdominelle Pathologien
Akute oder chronische Nierenschädigung mit Dialyse
Parenterale Ernährung

## Methodik

Eine Expertenrunde von Dermatologen mit langjähriger Erfahrung im Umgang mit Psoriasis und Mykosen und einem Mikrobiologen hat im Oktober 2023 in Berlin Handlungsanweisungen im Umgang mit diesen Patienten für den Praxisalltag erarbeitet. Grundlage waren die publizierte Evidenz in der S1-Leitlinie der Deutschsprachigen Mykologischen Gesellschaft (DMykG) und der Paul-Ehrlich-Gesellschaft für Chemotherapie (PEG) [5], der S3-Leitlinie zur Therapie der Psoriasis der Deutschen Dermatologischen Gesellschaft [7] sowie die klinisch-praktischen Erfahrungen der Autoren.

## Präventivmaßnahmen

Patienten, bei denen eine Biologikatherapie mit IL-17-Inhibitoren geplant ist, sollten bereits im Beratungsgespräch über das erhöhte Risiko für *Candida*-Infektionen aufgeklärt werden. Dabei steht die Aufklärung der Symptome im Vordergrund, sodass Infektionen früh erkannt werden und Betroffene sich zeitnah zwecks therapeutischer Maßnahmen vorstellen. Insbesondere in Risikogruppen sollten prophylaktische Maßnahmen zur Mundhygiene empfohlen werden, um das Risiko oraler Candidosen zu minimieren ([1]; Tab. 2). Dazu gehört auch eine regelmäßige professionelle Zahnreinigung (PZR). Aufgrund der essenziellen Bedeutung für die Mundhygiene kritisieren die Experten, dass die PZR nicht regelhaft von den gesetzlichen Krankenkassen übernommen wird und sich somit nicht alle Patienten diese regelmäßig leisten können.

**Tab. 2:** Maßnahmen zur Prophylaxe oraler Candidosen

Konsequente Mundhygiene: gutes Zähneputzen mit fluoridhaltiger Zahnpasta inklusive Reinigen der Zunge
Vermeidung von Mundtrockenheit: ausreichende Flüssigkeitszufuhr ggf. Kauen von zuckerfreiem Kaugummi zur Anregung der Speichelproduktion
Ausgewogene Ernährung: arm an Zucker und Kohlenhydraten
Verwendung von Mundspülungen, wie z. B. Chlorhexidin
Einnahme von Probiotika
Nikotinverzicht

## Handlungsempfehlungen bei klinischen Symptomen einer Candidose

**Abb. 2:**
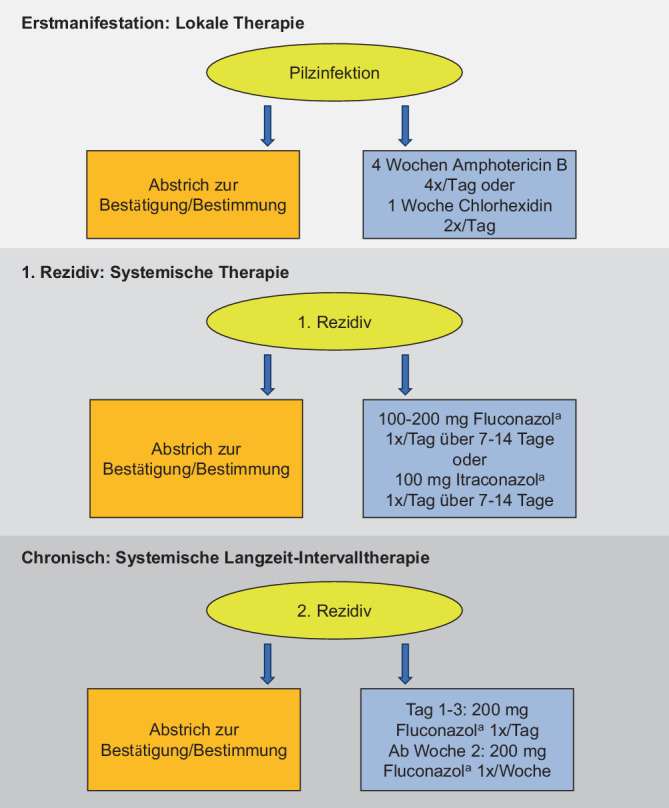
Therapiealgorithmus zum Vorgehen bei einer oralen Candidose. ^a^Kontraindikationen beachten

Wird der Patient mit einer klinisch symptomatischen Pilzinfektion vorstellig, sollte vor Beginn einer antimykotischen Therapie ein Abstrich für eine Pilzkultur entnommen werden, um die Spezies zu bestimmen. Bei klinischem Verdacht sollte die lokale antimykotische Therapie eingeleitet werden, ohne das Ergebnis des Abstrichs abzuwarten. Bei Erstmanifestation empfiehlt sich eine lokale Therapie mit Amphotericin B (Lutschtabletten oder Suspension 4‑mal täglich nach der Mahlzeit bzw. zur Nacht) über 4 Wochen oder Chlorhexidin (Suspension, 2‑mal täglich über 1 min) für eine Woche (Abb. 2). Bei einer Langzeitanwendung von Chlorhexidin kann es zu einer zumeist reversiblen Braunfärbung der Zähne kommen. Alternativ können auch Miconazol oder Nystatin angewandt werden.

## Handlungsempfehlungen beim Rezidiv

Das Auftreten von Rezidiven ist patientenabhängig und häufig Folge mangelnder Compliance. Bei Rauchern wurde ein höheres Risiko für Rezidive beobachtet. Hier sollte erneut ein Kontrollabstrich durchgeführt werden. Analog zur S3-Leitlinie zur Therapie der Psoriasis vulgaris wird ergänzend zu der oben genannten Lokaltherapie mit Amphotericin B oder Chlorhexidin eine systemische Therapie mit Fluconazol empfohlen. Dabei werden 100–200 mg Fluconazol 1‑mal täglich über 7 bis 14 Tage bis zur Remission eingenommen [7]. Alternativ kann mit Itraconazol 100 mg/Tag p.o. behandelt werden (Abb. 2). Als CYP3A4-Inhibitoren ist bei beiden Azolen die gleichzeitige Einnahme von Statinen aufgrund von Wechselwirkungen kontraindiziert. Bei Komedikation mit Statinen sollte daher eine hausärztliche oder internistische Rücksprache erfolgen. Das Risiko für Wechselwirkungen ist bei Itraconazol höher als bei Fluconazol.

## Chronische Candidose

Ab einem zweiten Rezidiv ist eine Candidose als chronisch anzusehen und kann eine systemische Langzeitintervalltherapie mit Fluconazol oder Itraconazol über ≥ 14 Tage erfordern, ggf. gefolgt von einer Erhaltungstherapie oder proaktiven Therapie. In der klinischen Praxis hat sich gezeigt, dass für rezidivierende orale Candidosen ein verkürztes Schema angelehnt an die Empfehlungen zur Therapie vulvovaginaler Candidosen ausreichend ist (Abb. 2; [6]). Hier empfiehlt sich Fluconazol 200 mg 1‑mal/Tag über 3 Tage, gefolgt von 200 mg 1‑mal/Woche.

## Fazit für die Praxis

- Bei einigen Patienten mit entzündlichen Erkrankungen können unter einer Anti-IL-17-Antikörper-Therapie *Candida*-Infektionen auftreten.
- Sowohl für Dermatologen als auch Patienten ist es wichtig, Symptome einer Candidose zu erkennen und Behandlungsoptionen zu kennen:
  a. bei Erstmanifestation: lokale Therapie,
  b. beim 1. Rezidiv: systemische Therapie,
  c. ab dem 2. Rezidiv: systemische Langzeitintervalltherapie.
- Bei einer wirksamen Behandlung der Candidose kann die Psoriasistherapie mit dem Biologikum fortgeführt werden.

## Interessenkonflikt

K. Meier: AbbVie Deutschland GmbH & Co. KG, Amgen GmbH, Almirall Hermal GmbH, AstraZeneca, Biogen GmbH, Bristol-Myers Squibb GmbH & Co. KGaA, Lilly Deutschland GmbH, Janssen-Cilag GmbH, UCB Pharma GmbH, Leo Pharma, Novartis Pharma GmbH, Takeda Pharmaceutical. S. Beissert: Advisory Board: AbbVie Deutschland GmbH & Co. KG, Actelion Pharmaceuticals Deutschland GmbH, AMGEN GmbH, Celgene GmbH, Galderma Laboratorium GmbH, Janssen-Cilag GmbH, LEO Pharma GmbH, Lilly Deutschland GmbH, Novartis Pharma GmbH, MSD SHARP & DOHME GMBH, Menlo Therapeutics, Sanofi-Aventis Deutschland GmbH, Pfizer Pharma GmbH, UCB Pharma GmbH; Speaker honorarium: Novartis Pharma GmbH, AbbVie Deutschland GmbH & Co. KG, MSD SHARP & DOHME GMBH, Pfizer Pharma GmbH, Janssen-Cilag GmbH, Galderma Laboratorium GmbH, Celgene GmbH, LA ROCHE-POSAY LABORATOIRE PHARMACEUTIQUE, Actelion Pharmaceuticals Deutschland GmbH, GlaxoSmithKline GmbH & Co. KG, Bristol-Myers Squibb GmbH & Co. KGaA, Sanofi-Aventis Deutschland GmbH, Almirall Hermal GmbH, Sandoz/HEXAL AG, Incyte Biosciences Germany GmbH; Clinical Trials: Janssen-Cilag GmbH, AMGEN GmbH, AbbVie Deutschland GmbH & Co. KG, Celldex Therapeutics, Aristea Therapeutics, Celgene GmbH, Galderma Laboratorium GmbH, Kiniksa Pharmaceuticals, LEO Pharma GmbH, DICE Therapeutic Inc., Lilly Deutschland GmbH, Novartis Pharma GmbH, MSD SHARP & DOHME GMBH, Menlo Therapeutics, Numab Therapeutics AG, Sanofi-Aventis Deutschland GmbH, Pfizer Pharma GmbH, UCB Pharma GmbH, F. Hoffmann-La Roche Ltd, DS Biopharma. K. Ghoreschi: erhielt Honorare für Vorträge oder Beratungen von folgenden Firmen: AbbVie, Almirall, Boehringer Ingelheim, Bristol-Myers Squibb, Celltrion, Eli Lilly, Janssen-Cilag, Leo Pharma, Novartis Pharma, Pfizer und UCB Pharma. S. Philipp: Vortrags‑/Beraterhonorare, klinische Studien: AbbVie, Almirall, Amgen, Biogen, BMS GmbH, Boehringer-Ingelheim, Eli Lilly, GSK, Hexal, Janssen Cilag, Klinge Pharma, Leo Pharma, MSD, Novartis, Pfizer, UCB Pharma. B. Schwarz: AbbVie, Almirall, Amgen , Biogen, Bristol-Meyers-Squibb, Dermapharm, Ferrer, Galderma, Incyte, Janssen, Leo Pharma, Lilly Pharma, Novartis, Pfizer, Regeneron, Sanofi, UCB in Form von Studien, Vorträgen und/oder Beratertätigkeit. H.-J. Tietz: führt Vorträge und Seminare durch, mit Unterstützung von: Bayer, Pierre Fabre, ISDIN, EUROIMMUN, UCB, Janssen, AbbVie. K. Asadullah: kooperierte und erhielt finanzielle Zuwendungen von den folgenden Firmen: AbbVie, Almirall, Antabio, Bayer, Celltrion, Bristol-Myers Squibb, Euroimmune, Emphasis, Emeriti Pharma, Galderma, Janssen, La Roche-Posay, LEO, L’Oréal, Novartis, Parexel International, Pierre Fabre, Roxall, RG, Sanofi Genzyme, TFS Trial Form Support, UCB.
